# Supplementary material for: Community Strategy for Hepatitis B, C, and D Screening and Linkage to Care in Mongolians Living in Spain
Source: Viruses. 2023 Jul 5;15(7):1506. doi: 10.3390/v15071506 (PMC10384786; doi:10.3390/v15071506)
Supplement: Supplementary file 1 [file viruses-15-01506-s001.zip › viruses-2416127-supplementary.pdf]

Supplementary Figure 1. Knowledge questionnaire translated to Mongol.

Элэгний гепатитийн мэдлэгийн санал асуулга

1. Гепатит гэдэг нь элэгний үрэвсэл

   Тийм *Үгүй*

2. Гепатит нь вирусээр үүсгэгдэг

   Тийм *Үгүй*

3. Гепатиттай байхад бие эрүүл мэт байж болох уу

   Тийм *Үгүй*

4. Гепатит нь архаг хүнд хэлбэртэй байж болох уу

   Тийм *Үгүй*

5. Гепатит В нь бэлгийн замаар дамжих боломжтой

   Тийм *Үгүй*

6. Гепатит С нь хувийн ариун цэврийн хэрэглэлийг хуваалцвал халдварлахболомжтой

   Тийм *Үгүй*

7. Гепатитийн зарим төрлийн эсрэг вакцин байдаг

   Тийм *Үгүй*

8. Гепатитийн вирус илрүүлэх шинжилгээ байдаг

   Тийм *Үгүй*
